# Unexpected Modulation of Recall B and T Cell Responses after Immunization with Rotavirus-like Particles in the Presence of LT-R192G

**DOI:** 10.3390/toxins2082007

**Published:** 2010-08-05

**Authors:** Fatou Thiam, Cyrille Di Martino, Fabienne Bon, Annie Charpilienne, Claire Cachia, Didier Poncet, John D. Clements, Christelle Basset, Evelyne Kohli

**Affiliations:** 1 Laboratoire des Interactions Muqueuses-Agents transmissibles (LIMA), UPR562, UFRs Médecine et Pharmacie, IFR Santé-STIC, Université de Bourgogne, Dijon, France; Email: thiam.fatou@hotmail.fr (F.T.); cyrilledimartino@yahoo.fr (C.D.M.); Fabienne.Bon@u-bourgogne.fr (F.B.); ccachia@u-bourgogne.fr (C.C.); evelyne.kohli@u-bourgogne.fr (E.K.); 2 Virologie Moléculaire et Structurale, UMR CNRS 2472 INRA 1157, Gif/Yvette, France; Email: annie.charpilienne@vms.cnrs-gif.fr (A.C.); didier.poncet@vms.cnrs-gif.fr (D.P.); 3 Department of Microbiology and Immunology, Tulane University Health Sciences Center, New Orleans, LA 70112, USA; Email: jclemen@tulane.edu (J.D.C.)

**Keywords:** LT-R192G, Foxp3, CD25, regulatory T cells, B lymphocyte, B-1a lymphocyte, mucosal immunization, rotavirus

## Abstract

LT-R192G, a mutant of the thermolabile enterotoxin of *E. coli*, is a potent adjuvant of immunization. Immune responses are generally analyzed at the end of protocols including at least 2 administrations, but rarely after a prime. To investigate this point, we compared B and T cell responses in mice after one and two intrarectal immunizations with 2/6 rotavirus-like particles (2/6-VLP) and LT-R192G. After a boost, we found, an unexpected lower B cell expansion measured by flow cytometry, despite a secondary antibody response. We then analyzed CD4^+^CD25^+^Foxp3^+^ regulatory T cells (Tregs) and CD4^+^CD25^+^Foxp3^−^ helper T cells after *in vitro* (re)stimulation of mesenteric lymph node cells with the antigen (2/6-VLP), the adjuvant (LT-R192G) or both. 2/6-VLP did not activate CD4^+^CD25^+^Foxp3^−^ nor Foxp3^+^ T cells from non-immunized and 2/6-VLP immunized mice, whereas they did activate both subsets from mice immunized with 2/6-VLP in the presence of adjuvant. LT-R192G dramatically decreased CD4^+^CD25^+^Foxp3^+^ T cells from non-immunized and 2/6-VLP immunized mice but not from mice immunized with 2/6-VLP and adjuvant. Moreover, in this case, LT-R192G increased Foxp3 expression on CD4^+^CD25^+^Foxp3^+^ cells, suggesting specific Treg activation during the recall. Finally, when both 2/6-VLP and LT-R192G were used for restimulation, LT-R192G clearly suppressed both 2/6-VLP-specific CD4^+^CD25^+^Foxp3^−^ and Foxp3^+^ T cells. All together, these results suggest that LT-R192G exerts different effects on CD4^+^CD25^+^Foxp3^+^ T cells, depending on a first or a second contact. The unexpected immunomodulation observed during the recall should be considered in designing vaccination protocols.

## 1. Introduction

Because systemic immunization is not optimal to induce local immune effectors and requires multiple injections, mucosal immunization is an important goal of vaccine development to protect against pathogens that use mucosa as portals of entry. However, several factors such as antigen degradation in the digestive tract, compartmentalization of the responses at the mucosa of antigen delivery as well as mucosal tolerance make mucosal vaccination with non replicating antigens complex. To generate strong mucosal immune responses, mucosal adjuvants have been proposed. The cholera toxin, CT, and the thermolabile enterotoxin of *Escherichia coli*, LT, which are well known diarrheagenic toxins produced by V. *cholerae* and enterotoxinogen *E. coli*, respectively, are potent mucosal adjuvants for abrogating mucosal tolerance and inducing strong B and T cell immune responses against coadministered antigens. To overcome the enterotoxicity and use them as adjuvant in humans, non toxic mutants of the A subunit have been developed, among them, the protease site mutant of LT, LT-R192G. This mutant retains adjuvant properties in experimental models [1,2,3,4] and has been tested in clinical trials [5,6,7]. However, although many studies have reported about the effects of the whole toxins or their mutants on different innate or adaptive immune cells that could explain the adjuvant effect (reviewed in [1,2,3,4,8,9]), the precise mechanism of action of these adjuvants has not been completely elucidated. Of note, the comparison of their different effects on immune responses after a prime and a boost, using the same route and the same immunogen, has not been documented. This comparison may bring relevant cognitive information and, in addition, it may be useful to optimize protocols of immunization. 

To better understand early events induced after mucosal priming with a non-replicating antigen, we previously traced rotavirus (RV)-specific B cells by flow cytometry (FCM), after a single intranasal (IN) or intrarectal (IR) administration of RV virus-like particles (2/6-VLP) in mice, in the presence of LT-R192G [10,11]. 2/6-VLP coadministered intranasally or intrarectally with LT-R192G, in protocols including at least 2 immunizations, have been shown to induce strong T and B cell responses as well as protection against experimental infection [12,13]. With both routes, we have shown, after a prime, high expansion of specific B cells in different lymphoid tissues, which depend on the route of immunization. A substantial proportion of these cells expressed CD5 and was considered B-1a cells. Unexpectedly, we found that a second IN immunization in the same conditions did not increase the frequency of specific B cells on day 7 following the second immunization, whereas a secondary systemic and mucosal antibody response was observed [11]. As LT-R192G is a potent mucosal adjuvant, this result was therefore difficult to explain. We hypothesized that the massive B cell expansion observed when the adjuvant was used for immunization was probably regulated during the second contact. Such modulation may be important to avoid potential deleterious autoreactivity of CD5^+^ expressing B-1a cells, as well as T cell-mediated inflammation. Regulatory T lymphocytes (Tregs) are important in suppressing the activation, proliferation and differentiation of T and B cells, and thus control immune responses [14,15]. Classically, Tregs are divided into two major subtypes: natural Tregs (nTregs) and peripherally inducible Tregs (iTregs). CD4^+^ nTregs develop in the thymus and express CD25 and the forkhead box P3 transcription factor, Foxp3 [16]. CD4^+^ iTregs include different subtypes, among which is a subpopulation that shares the same phenotype, CD25^+^Foxp3^+^, with nTregs [17]. In mice, Foxp3 does not make it possible to distinguish between nTregs and iTregs but is considered a useful marker to distinguish between CD4^+^ T cells with a presumed regulatory-suppressive function and other CD4^+^ T cells [18]. 

In this work, to investigate more in details the effects of LT-R192G after a prime and a boost, we first compared the primary and secondary specific B cell response induced by IR immunization with 2/6-VLP in the presence or in the absence of LT-R192G. Then, we analyzed specific CD25^+^CD4^+^ T cells, both Foxp3^+^ and Foxp3^−^, from different lymphoid tissues, in *in vitro* response to 2/6-VLP, LT-R192G or both. Quantitative analysis reflecting activation and/or proliferative responsiveness was performed using cell frequency, CD25 and/or Foxp3 mean fluorescence intensity of both subsets, as well as IL-2 production [19]. 

## 2. Materials and Methods

### 2.1. Mice

Pathogen-free, adult, female BALB/c mice (6–8 weeks of age) were obtained from Iffa-Credo (L’Arbresle, France) or from our in-house facilities. Study protocols were approved by the local institutional animal care committee. No mouse had evidence of previous RV infection, as determined by serum antibody titres.

### 2.2. VLP Preparation and Adjuvant

Two different VLP, containing RV VP2 and VP6 (2/6-VLP) used for immunization or GFP-Δ92VP2 and VP6 used for flow cytometry, were produced in the baculovirus system as described previously [20]. Briefly, Sf9 insect cells were coinfected with two recombinant baculoviruses expressing the protein VP6 of the bovine RF strain and an authentic or a modified GFP-VP2 at a multiplicity of infection higher than 5PFU/cell. VLP were collected 5–7 days post infection and purified by density gradient centrifugation in CsCl.

LT-R192G, a LT mutant, the thermolabile enterotoxin of *Escherichia coli*, was used as the adjuvant. LT-R192G is a mutant of LT containing a single amino acid substitution that alters the site of proteolytic cleavage within the region joining A1 and A2. This mutation is associated with the reduced ability to induce an accumulation of cAMP in cultured cells as well as reduced enterotoxicity in experimental animals and humans when compared to native LT [2].

### 2.3. Immunization and Sample Collection

The mice were intrarectally immunized on day 0 with either NaCl, 10 µg LT-R192G alone, 10 µg 2/6-VLP alone or mixed with 10 µg LT-R192G. Prior to immunization, the mice were anesthetized by intraperitoneal administration of a mixture of ketamine (80 mg/kg) and xylazine (16 mg/kg). An additional group of mice was given two doses of 10 µg 2/6-VLP with 10 µg LT-R192G, or 2/6-VLP or LT-R192G alone on day 0 and on day 14. The mice were killed at different time points post-immunization (2, 4, 7 or 14 days) and the different lymphoid tissues, rectal follicles (RF), lumbar lymph nodes (LLN), mesenteric lymph nodes (MLN), Peyer’s patches (PP) and spleen were removed. Serum and faecal samples were collected and stored at −20 °C.

### 2.4. Measurement of Rotavirus-Specific Antibodies in Serum and Fecal Samples

Antibody titres in serum and faecal samples were determined by ELISA. Microtitre plates were coated overnight at 4 °C with 100 ng of 2/6-VLP in 100 µL of 0.1 M carbonate/bicarbonate buffer, pH 9.6. The wells were blocked with PBS containing 5% non-fat dry milk for 45 min at 37 °C. Faecal samples were made 10% (wt/vol) by suspension in PBS, pH 7.4. Serial twofold dilutions in PBS-5% non-fat dry milk of serum (starting at 1/100) or faecal extracts (starting at 1/40) were added to the wells and incubated for 40 min at 37 °C. After three washes with PBS-0.05% Tween 20, the plates were incubated for 30 min at room temperature with 1/5000 dilution of biotin-labelled goat anti-mouse IgA, IgG or IgM (Cell Lab, Beckman Coulter). The plates were washed, and 1/4000 of peroxidase-labeled avidin (Cell Lab, Beckman Coulter) was added for 30 min at room temperature. The colour reaction was developed with TMB-Peroxidase Substrate Kit (AbCys.S.A), stopped with 100 µL of H_2_SO_4_ 0.4N and A_450_ was determined. A sample was considered positive if the optical density of the sample well was >0.1 plus the mean of the OD values of the negative control wells. Titres were defined by the inverse of the highest serum dilution giving a positive signal. Negative serum and fecal samples (titre < 100 and <40, respectively) were arbitrarily assigned titres of 50 and 20 (half of 100 and 40), respectively, for statistical calculations [21].

### 2.5. Preparation of Cells from RF, LLN, MLN, PP and Spleen

Single-cell suspensions were prepared by mechanical dissociation, filtered on 40-µm-pore nylon meshes and washed with incomplete medium (RPMI-1640 supplemented with 0.3% glucose, 100 U penicillin per mL, and 100 µg streptomycin per mL). The cells were counted, labelled and analyzed immediately by FCM or resuspended (4 × 10^6^/mL) in complete medium (incomplete medium plus 10% heat-inactived FCS, 2 mM L-glutamine, 1 mM sodium pyruvate) for *in vitro* restimulation.

### 2.6. *In Vitro* Restimulation

Cells from either immunized or non-immunized mice (4 × 10^5^ cells/well) were cultured in 96-well plates in the presence of different doses of 2/6-VLP, LT-R192G, 2/6-VLP and LT-R192G or RPMI medium only. The T-cell mitogen concanavalin A (5 µg/mL) was used as the positive control. The cells were incubated at 37 °C with 5% CO_2_ and harvested on day 2 and day 4 after restimulation for flow cytometry analysis, and the culture supernatants were frozen at −40 °C until IL-2 assay. 

### 2.7. FCM Assays

#### 2.7.1. Rotavirus Specific B Cell Quantification

To detect RV-specific B cells, we used an FCM assay as previously described [10]. Cells from different lymphoid tissues were washed once with PBS 1% BSA 0.1% sodium azide buffer. Pellets containing 2 × 10^6^ cells were incubated with a mixture of PE Cy-chrome-labelled anti-B220 (Clone RA3-6B2, Pharmingen, San Diego, CA, USA), biotinylated anti-IgD (Clone AM9.1, Pharmingen), PE-labelled anti-CCR9 (Clone 248918, R&D Systems, Minneapolis, MN, USA) or anti-CD5 (Clone 53-7.3, Pharmingen) or anti-α4β7 (clone DATK32, Pharmingen) and GFP-2/6-VLP (to detect RV-specific B lymphocytes) for 30 min in the dark at 4 °C. The cells were washed and then labelled with streptavidine-RED613 (Gibco-BRL, Scotland, UK). After incubation for 30 min in the dark at 4 °C, the cells were washed, resuspended in buffer containing PBS 1% BSA 0.1% sodium azide, and analysed on a flow cytometer (LSR II, Becton Dickinson, San Jose, CA, USA). Approximately 3 × 10^5^ cells were acquired. Analysis was done as described previously [10]. Small lymphocytes were distinguished from large lymphocytes by their light-scatter profile. Three types of B cell subsets were analysed: a large B220^int^ IgD^−^ subset representing extrafollicular B cells, a large B220^high^ IgD^−^ subset representing germinal center B cells, and a small B220^high^ IgD^−^ subset consisting of memory and germinal center B cells [10]. To delineate RV-specific cell populations and to control for the specificity of the staining, the cells were stained, omitting GFP-VLP, and the quadrant position was fitted eventually, after comparison with identical B cell subsets from non-immunized mice. Absolute numbers of RV-specific B cells were obtained by the quantification of RV-specific B cells among total cells that expressed B220 (B220^high^ plus B220^low^). In order to compare samples, the number of RV-specific B cells was referred to 10^5^ total B220^+^ cells [11].

#### 2.7.2. Analysis of CD4^+^CD25^+^Foxp3^+^ T Cells and CD4^+^CD25^+^Foxp3^−^ T Cells

CD4^+^CD25^+^Foxp3^+^ T cells and CD4^+^CD25^+^Foxp3^−^ T cells were quantified using the Mouse Regulatory T cell Staining kit #2 (w/APC Foxp3 FJK-16s, FITC CD4, PE CD25; eBioscience, San Diego, USA). Briefly, pellets containing 4x10^5^ cells in 100 µL PBS 1% BSA 0.1% sodium azide, were incubated with a mixture of FITC-labelled anti-CD4 and PE-labelled anti-CD25 for 30 min in the dark at 4 °C. The cells were washed and resuspended in 1 mL of freshly prepared fixation/permeabilization working solution and then incubated at 4 °C for 30 min in the dark. The cells were washed with permeabilization buffer and then labelled with APC-labelled anti-Foxp3 for 30 min in the dark. After incubation, the cells were washed, resuspended in buffer containing PBS 1% BSA 0.1% sodium azide, and analysed by flow cytometry. Approximately 5 × 10^4^ cells were acquired. Lymphocytes were first identified by their light-scatter profile, and T cell subsets were then identified by CD4 expression. CD25^+^Foxp3^+^ and CD25^+^Foxp3^−^ T cells were identified by binding of CD25 and Foxp3 within CD4^+^ T cells. The mean fluorescence intensity (MFI) for PE and APC were analyzed to detect variations in CD25 and Foxp3 expression, respectively. 

### 2.8. IL-2 Assay

The IL-2 level in culture supernatants was determined by ELISA, using the AbC601MST Mouse IL-2 Module Set kit (AbCys.S.A). Briefly, microtitre plates were coated with 100 µL of anti-IL-2 monoclonal antibody and incubated overnight at 4 °C. The wells were blocked with PBS containing 0.5% BSA and 0.05% Tween 20 at 4 °C overnight. After two washes with PBS containing 0.05% Tween 20, 50 µL of supernatants were added and incubated with the biotinylated anti-IL-2 monoclonal antibody for 2h. The plates were washed, peroxidase-labeled streptavidine was added and the plates were incubated for 1h. The colour reaction was developed with TMB-Peroxidase Substrate Kit (AbCys.S.A), stopped with 100 µL of H_2_SO_4_ 0.4 N and A_450_ was determined.

### 2.9. Statistics

For B cell analysis, results were expressed as means with SEM. Statistical analysis was performed using SigmaStat software. Pairwise comparisons between non-immunized and immunized mice were made using the Mann-Whitney non-parametric U-test. P-values less than 0.05 were considered statistically significant. 

For T cell analysis, comparison of cell frequency, CD25 and Foxp3 MFI between stimulated and non stimulated wells was done by using the Wilcoxon paired non-parametric signed-rank test. P-values less than 0.05 were considered statistically significant. In addition, comparisons between non-immunized and immunized mice were made using the Mann-Whitney unpaired non-parametric U-test.

## 3. Results

### 3.1. Primary and Secondary Specific B Cell Responses Induced by IR Immunization with 2/6-VLP with or without LT-R192G

#### 3.1.1. The Secondary Response Induced by IR Immunization with 2/6-VLP and LT-R192G Showed a Serum and Fecal Antibody Response but a Lower RV-Specific B Cell Expansion

**Figure 1:**
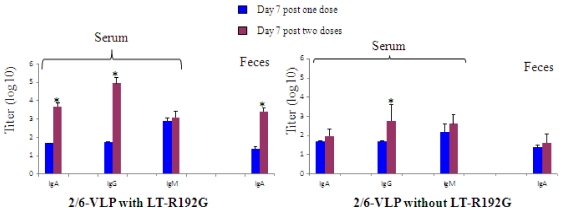
RV-specific antibody responses in serum and feces from BALB/c mice IR immunized with 2/6-VLP alone or with LT-R192G on day 7 post one (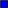) or two (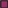) immunizations. The results are plotted as the geometric mean titres (n = 5). * Data points statistically different between one and two doses (p < 0.05).

Mice were immunized once or twice with 2/6-VLP or NaCl in the presence of adjuvant, and the RV-specific B cell response analyzed as described previously [10]. On day 7 after the second dose, a secondary antibody response was observed in serum (IgA 3.7 *vs*. 1.7 log and IgG 5 *vs*. 1.7 log, for the secondary and primary response, respectively) and in feces (IgA 3.4 *vs*. 1.4 log)(Figure 1), as already shown with the IN route of immunization [11].

**Figure 2:**
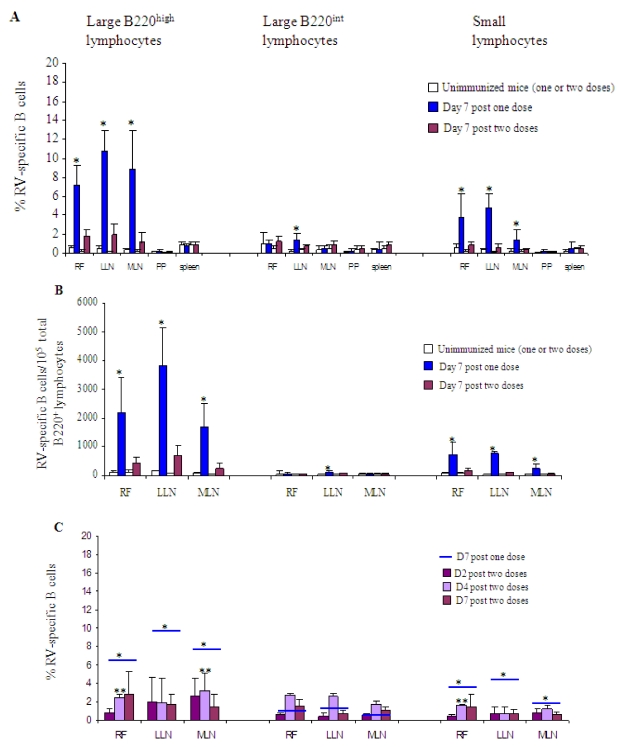
Primary and secondary specific B cell response induced by IR immunization with 2/6-VLP and the mucosal adjuvant LT-R192G. (A) Percentage of RV-specific B cells in FR, LLN, MLN, PP and spleen on day 7 post one (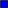) or two (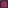) immunizations with 2/6-VLP and LT-R192G. The results are plotted as the means, and error bars represent 1 SEM (n = 7). * Data points statistically different between one and two doses (p < 0.05). Analysis was done as previously described [10]. Briefly, small lymphocytes were distinguished from large lymphocytes by their light-scatter profile. Three types of B cell subsets were analysed: the large B220^int^ IgD^−^ B cell subset representing extrafollicular B cells, the large B220^high^ IgD^−^ B cell subset representing germinal center B cells, and the small B220^high^ IgD^−^ lymphocyte subset consisting of memory and germinal center B cells. (B) Frequency (RV-specific B cells/10^5^ total B220^+^ cells) of RV-specific B cells in RF, LLN and MLN on day 7 post one (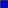) or two (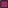) immunizations with 2/6-VLP and LT-R192G. The results are plotted as the means, and error bars represent 1 SEM (n = 7). * Data points statistically different between one and two doses (p < 0.05). (C) Percentage of RV-specific B cells in RF, LLN and MLN after two immunizations with 2/6-VLP and LT-R192G on d2 (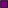), d4 (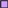) and d7 (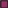). On day 7 after one immunization, the results obtained are represented by 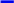. The results are plotted as the means, and error bars represent 1 SEM (n = 3–7). * Data points statistically different between one and two doses on day 2, 4 or 7 (p < 0.05). ** Data points statistically different between one (data not shown) and two doses on day 4 (p < 0.05).

A RV-specific B cell response, measured by flow cytometry using GFP-2/6-VLP, was found on day 7 in RF, LLN and MLN, but not in PP and the spleen, as previously reported after a single immunization (Figure 2A) [10]. However, the secondary B cell response was significantly lower than the primary response within the large B220^high^ B cells (1.5–2% *vs*. 7–11% depending on the lymphoid tissue) and the small lymphocytes (0.4–1% *vs*. 1.5–5%)(Figure 2A). This result was confirmed when the B cell frequency was expressed as absolute numbers of RV-specific B cells/10^5^ total B220^+^ cells (Figure 2B). For the large B220^int^ lymphocyte subset, no major difference was observed between the primary and the secondary response, but in this case, no massive expansion was observed during the primary response (Figure 2B). As the lower frequency observed could be the consequence of an earlier expansion during the secondary response, we further analyzed the B cell response on day 2 and 4 after a second dose and on day 4 after a single dose (Figure 2C). The results clearly confirmed the absence of a massive B cell expansion during the secondary response, despite a trend towards a higher response on day 4 for the B220^int^ subset, which is consistent with the kinetics of a secondary antibody response. 

Of note, α4β7, CCR9 and CD5 expression by RV-specific B cells was not different in the primary and secondary responses (<15%, 30–50% and 30–75%, respectively, data not shown).

All together, these results show that, after two immunizations with 2/6-VLP and LT-R192G, the massive expansion observed during the primary response was no longer observed during the secondary response despite a secondary antibody response.

#### 3.1.2. The Secondary Response Induced by IR Immunization with 2/6-VLP in the Absence of Adjuvant Showed a Similar RV-Specific B Cell Expansion to that in the Primary Response and a Secondary Serum IgG Antibody Response

When mice were immunized with 2/6-VLP in the absence of adjuvant, a significant IgG antibody response was observed on day 7 after two immunizations (Figure 1). Concerning the RV-specific B cell response, we have previously shown a great variability among mice after one immunization with 2/6-VLP alone. Furthermore, when no adjuvant was used, we only observed a response in LLN. After two immunizations, a very similar response was observed in terms of heterogeneity and intensity (6 *vs*. 5.2% in B220^high^ large B cells, 1.5 *vs*. 1% in B220^int^ cells and 1.8 *vs*. 1.8% in small lymphocytes, on day 7 following the first and second immunization in LLN, data not shown). In addition, as after one immunization, no significant response was observed in MLN after two immunizations in the absence of adjuvant.

### 3.2. Primary and Secondary *in Vitro* T Cell Responses to 2/6-VLP, LT-R192G or Both, from Non-Immunized Mice and Mice Immunized with 2/6-VLP with or without LT-R192G: Analysis of CD4^+^CD25^+^Foxp3^−^ and CD4^+^CD25^+^Foxp3^+^ T Cells

Non-immunized mice and mice immunized with 2/6-VLP, with or without LT-R192G, were sacrificed on day 14 and cells from different lymphoid tissues (4 × 10^5^ cells/well) were cultured in the presence of antigen (5 µg/mL), adjuvant (5 µg/mL) or both for 4 days. CD4^+^CD25^+^Foxp3^−^ and CD4^+^CD25^+^Foxp3^+^ T cells were analyzed by flow cytometry and IL-2 was quantified in culture supernatants. Results were similar for all the organs or tissues analyzed (*i.e.*, LLN, MLN, PP and the spleen), but as they contain a high number of cells allowing multiple wells for *in vitro* culture, we focussed on MLN and performed statistical analysis only for MLN. RF were not analyzed because the number of cells harvested was not sufficient for *in vitro* restimulation. 

#### 3.2.1. Concanavalin A Activates CD4^+^CD25^+^Foxp3^−^ and CD4^+^CD25^+^Foxp3^+^ T Cells from All Mice

**Figure 3:**
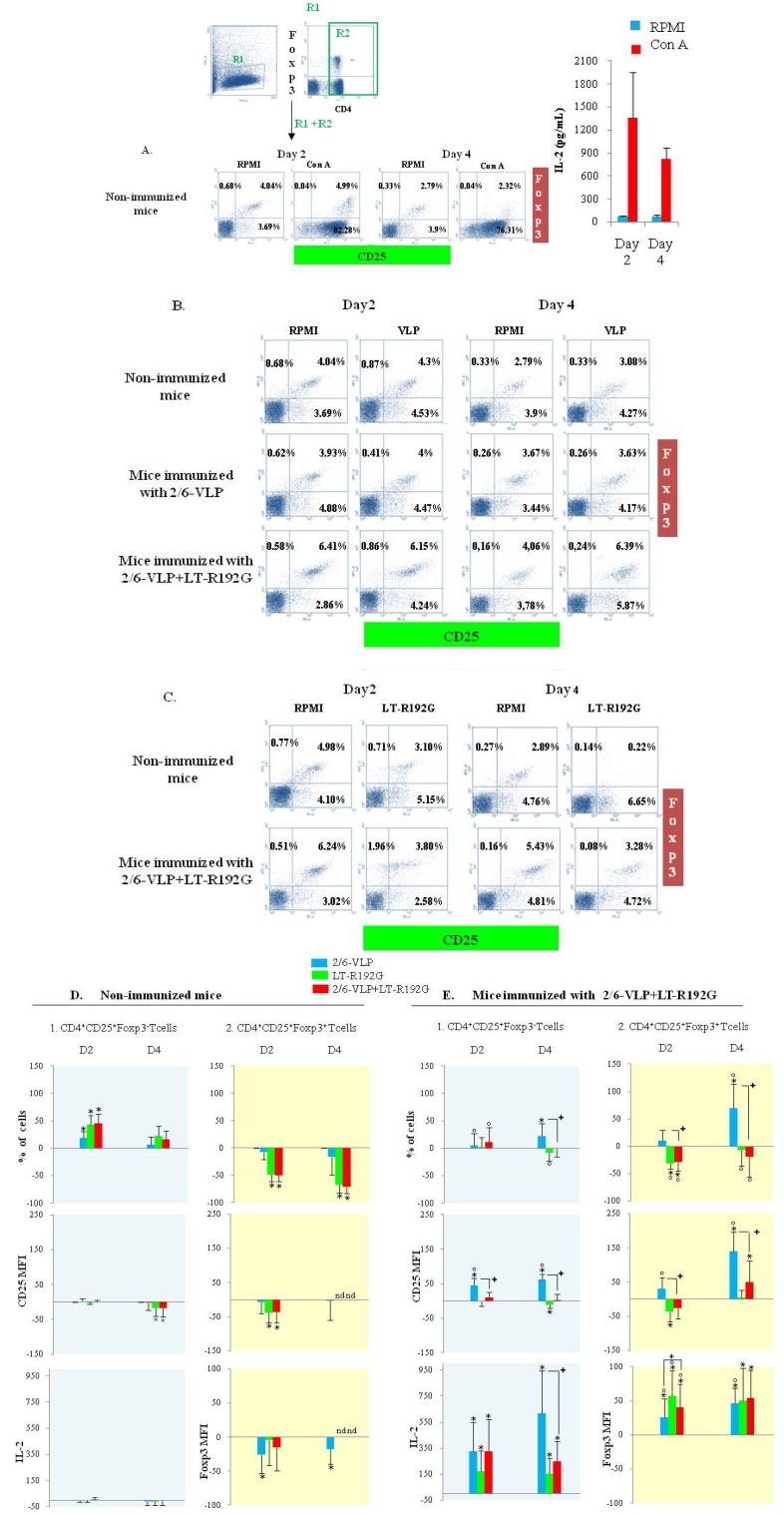
Primary and secondary *in vitro* T cell responses to 2/6-VLP, LT-R192G or both from non-immunized mice and mice immunized with 2/6-VLP with or without LT-R192G. Micewere sacrificed on day 14 after one immunization and cells from MLN (4 × 10^5^ cells/well) were cultured in the presence of Concanavalin A (5 μg/mL), antigen (5 μg/mL), adjuvant (5 μg/mL) or both for 2 and 4 days. Percentage of CD4^+^CD25^+^Foxp3^−^ and CD4^+^CD25^+^Foxp3^+^ T cells and both CD25 and Foxp3 MFI were analyzed by flow cytometry and IL-2 was quantified in culture supernatants. Lymphocytes were first identified in a dotplot from light-scatter, and T cell subsets were then identified by expression of CD4. CD25^+^Foxp3^+^ and CD25^+^Foxp3^−^ T cells were then identified by binding of anti-CD25 and anti-Foxp3 antibodies within CD4^+^ T cells. (A) Stimulation with Concanavalin A and quantification of IL-2 in non-immunized mice. (B)(Re) stimulation with antigen (5 μg/mL) in non-immunized mice and mice immunized with 2/6-VLP with or without LT-R192G. (C)(Re) stimulation with LT-R192G (5μg/mL) in non-immunized mice and mice immunized with 2/6-VLP and LT-R192G. These dotplots of one experiment are representative of 7 separate experiments. (D) and (E): The histograms show the percentage of increase or decrease for each parameter (2/6-VLP (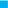) or LT-R192G (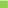) or both (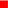)) compared to the control well (RPMI). (D) Non-immunized mice. (E) Immunized mice. Data represent mean ± SEM of six or seven separate experiments. P-values were calculated using the Wilcoxon paired non-parametric signed-rank test or the Mann-Whitney unpaired non-parametric U-test. * Data points statistically different between stimulated and RPMI wells (p = 0.01, 0.015 or 0.025). + Data points statistically different between 2/6-VLP and 2/6-VLP and LT-R192G stimulated wells. Percentages of increase or decrease compared to the control RPMI statistically different between non-immunized mice and immunized mice (p < 0.01 or p ≤ 0.05). nd: not determined.

In order to check cell viability and validate our *in vitro* model, we stimulated cells from immunized and from control mice with concanavalin A. As expected, an increase in CD4^+^CD25^+^Foxp3^−^ cell frequency, associated with considerable production of IL-2 was observed (Figure 3A), whereas CD4^+^CD25^+^Foxp3^+^ cell frequency was stable despite a major increase in CD25 expression reflecting cell activation.

#### 3.2.2. Analysis of CD4^+^CD25^+^Foxp3^−^ and CD4^+^CD25^+^Foxp3^+^ T Cells from Non-Immunized Mice after *in Vitro* Culture with 2/6-VLP or LT-R192G or Both

*In vitro* culture without restimulation (RPMI medium) showed a decrease in CD4^+^CD25^+^Foxp3^+^ T cell frequency from day 2 to day 4 (Figure 3B and Figure 3C) as previously reported [14].

*In vitro* culture with 2/6-VLP. As expected, *in vitro* culture in the presence of 2/6-VLP did not activate CD4^+^CD25^+^Foxp3^−^ T cells from non-immunized mice as shown by the stability of CD25 and the lack of IL-2 production (Figure 3B and Figure 3D1). No increase in CD25 or in Foxp3 expression by CD4^+^CD25^+^Foxp3^+^ cells was observed in the presence of 2/6-VLP (Figure 3B and Figure 3D2). Of note, on day 2, there was a weak increase in CD4^+^CD25^+^Foxp3^−^ cell percentage (p = 0.01) going with a weak decrease of CD4^+^CD25^+^Foxp3^+^ cell percentage. This could be explained by a decrease of Foxp3 expression on day 2 and 4 (p = 0.01)(Figure 3D).

*In vitro* culture with LT-R192G. As observed with 2/6-VLP, LT-R192G did not activate CD4^+^CD25^+^Foxp3^−^ nor CD4^+^CD25^+^Foxp3^+^ T cells from non-immunized mice as shown by the lack of increase of CD25 and Foxp3 MFI and the lack of IL-2 production (Figure 3C and Figure 3D). At the opposite and unlike 2/6-VLP, a significant decrease of CD25 expression on both CD4^+^CD25^+^Foxp3^−^ (p = 0.01 on day 4) and CD4^+^CD25^+^Foxp3^+^ cells (p = 0.01 on day 2) was observed. The decrease in CD25 expression on CD4^+^CD25^+^Foxp3^+^ cells was associated with a major decrease of their frequency from day 2 (p = 0.01). An increase in the frequency of CD4^+^CD25^+^Foxp3^−^ cells was also observed, notably on day 2 (p = 0.01), suggesting that a part of CD4^+^CD25^+^Foxp3^+ ^ T cells did not express Foxp3 anymore and became Foxp3^−^. Of note, on day 4, CD25 and Foxp3 expression was not analyzed because of the very low number of remaining CD4^+^CD25^+^Foxp3^+^ cells.

*Dose-effect*. When testing different doses of LT-R192G, we observed that a dose of 0.01 µg/mL was sufficient to induce a decrease in CD25 expression by CD4^+^CD25^+^Foxp3^+^ T cells (about 25%), suggesting the efficacy of this molecule. The maximum was observed at doses of 0.5 and 1 µg/mL (about 70%)(data not shown).

*In vitro* culture with both 2/6-VLP and LT-R192G. When cells were cultured with both antigen and adjuvant, the results were similar to those observed with LT-R192G (Figure 3D).

#### 3.2.3 Analysis of CD4^+^CD25^+^Foxp3^−^ and CD4^+^CD25^+^Foxp3^+^ T Cells from Mice Immunized with 2/6-VLP Alone after *in vitro* Culture with 2/6-VLP or LT-R192G or Both

The results were similar to those observed with non- immunized mice (Figure 3B, data not shown).

#### 3.2.4 Analysis of CD4^+^CD25^+^Foxp3^−^ and CD4^+^CD25^+^Foxp3^+^ T Cells from Mice Immunized with 2/6-VLP and LT-R192G and Restimulated *in vitro* with 2/6-VLP or LT-R192G or Both

As for non-immunized mice and as expected, *in vitro* culture without restimulation (RPMI medium) showed a decrease in CD4^+^CD25^+^Foxp3^+^ T cell frequency from day 2 to day 4 (Figure 3B and Figure 3C).

*In vitro* culture with 2/6-VLP. As expected, 2/6-VLP did activate CD4^+^CD25^+^Foxp3^−^ T cells as shown by the increase in CD25 MFI as early as day 2 (p = 0.01) and the increase in cell frequency on day 4 (p = 0.01) as well as the production of IL-2 from day 2 (p = 0.005)(Figure 3B and Figure 3E1). Activation of CD4^+^CD25^+^Foxp3^−^ T cells was associated with an increase in Foxp3 expression by CD4^+^CD25^+^Foxp3^+^ T cells (p = 0.025 and 0.01 on day 2 and 4, respectively), followed on day 4 by an increase in CD25 MFI (p = 0.01) and cell frequency (p = 0.015)(Figure 3B and Figure 3E2). 

Moreover, we have also compared the effect of the (re)stimulation between non-immunized and 2/6-VLP and LT-R192G immunized mice and found that CD25 MFI (p < 0.01 and p < 0.05 on day 2 and 4) for both subsets, as well as cell frequency (p < 0.01 on day 4) and Foxp3 MFI (p < 0.01 on day 2 and 4) for CD4^+^CD25^+^Foxp3^+^ T cells, were statistically different between non-immunized and 2/6-VLP and LT-R192G immunized mice (Figure 3E).

Dose-effect. We further tested different doses of 2/6-VLP and showed activation of CD4^+^CD25^+^Foxp3^−^ T cells (increase in CD25 expression) on day 2 from the dose of 1 µg/mL and an increase in their frequency on day 4 at the doses of 5 and 10 µg/mL (data not shown), which was consistent with IL-2 production on day 2. Activation of CD4^+^CD25^+^Foxp3^+^ T cells (increase in CD25 expression) was observed on day 4 from the dose of 0.5 µg/mL (data not shown).

All together, these results suggest that CD4^+^CD25^+^Foxp3^+^ T cells are activated when IL-2 is produced, *i.e.*, when CD4^+^CD25^+^Foxp3^−^ T cells are activated [19]. This is observed only in mice immunized in the presence of adjuvant.

*In vitro* culture with LT-R192G. Whereas IL-2 was produced (p = 0.005 on day 2 and p = 0.01 on day 4), no increase in CD25 MFI was observed neither by CD4^+^CD25^+^Foxp3^−^ nor by CD4^+^CD25^+^Foxp3^+^ T cells (Figure 3C and Figure 3E). At the opposite, there was a decrease in CD25 expression on day 4 and on day 2, compared to RPMI well, for CD4^+^CD25^+^Foxp3^-^ (p = 0.025) and CD4^+^CD25^+^Foxp3^+^ T cells (p = 0.015), respectively, that was not statistically different from non immunized mice. However, unlike control mice (p < 0.01 on day 2), CD4^+^CD25^+^Foxp3^+^ T cells were activated as shown by the increase in Foxp3 expression (p = 0.01 on day 2 and p = 0.015 on day 4) compared to the well with RPMI medium. Foxp3 MFI was not associated with an increase in cell frequency, nevertheless, unlike control mice, CD4^+^CD25^+^Foxp3^+^ T cells did not decrease compared to the RPMI well on day 4 (p < 0.01), and despite a transient decrease on day 2 that was clearly explained by the decrease of CD25 expression (the cells became CD25^−^) (Figure 3C and Figure 3E2). This result suggests that CD4^+^CD25^+^Foxp3^+^ T cells were activated in response to LT-R192G and were resistant to LT-R192G-induced cell death unlike CD4^+^CD25^+^Foxp3^+^ cells from non- immunized mice.

Dose-effect. The maximum effect on CD25 expression was observed at the same doses as for control mice and the increase in Foxp3 expression was observed at the dose of 5 µg/mL on day 2 and at all the doses on day 4 (data not shown).

All together, these results show major effects of LT-R192G on CD4^+^CD25^+^Foxp3^+^ T cells *in vitro* which are different between non-immunized and mice immunized in the presence of adjuvant (*i.e.*, first and second contact with LT-R192G).

*In vitro* culture with both 2/6-VLP and LT-R192G. Compared to wells cultured in the presence of 2/6-VLP alone, we observed a decrease in CD4^+^CD25^+^Foxp3^−^ T cell activation in the presence of LT-R192G as shown by the decrease in CD25 MFI, cell frequency and IL-2 production (p = 0.01 on day 2 and 4, p = 0.015 on day 4 and p = 0.005 on day 4, respectively)(Figure 3E1). These effects were associated with a decrease in CD25 MFI and cell frequency of CD4^+^CD25^+^Foxp3^+^ T cells (p = 0.01 on day 2 and day 4, for both parameters). Regarding Foxp3, it was greater on day 2 (p = 0.025) but not different at day 4 (Figure 3E2).

These results show that CD4^+^CD25^+^Foxp3^−^ T cells were modulated *in vitro* in the presence of LT-R192G compared to antigen alone, which was confirmed by the fact that IL-2 production decreased on day 4 (Figure 3E1).

Dose-effect. The results were confirmed using different doses of 2/6-VLP and LT-R192G in the same wells (5/0.1, 5/1, 5/5, 1/1 and 10/1 for the concentrations of 2/6-VLP and LT-R192G, respectively)(Data not shown). 

All together, our results confirm that the presence of the adjuvant substantially modulates CD4^+^CD25^+^Foxp3^+^ T cells *in vitro*, and that the response is different in the first and second contact. Moreover, they show that the adjuvant controls both CD4^+^CD25^+^Foxp3^−^ and CD4^+^CD25^+^Foxp3^+^ specific T cell responses induced by the antigen.

## 4. Discussion

In this work, we have shown an unexpected lower RV-specific B cell expansion measured by flow cytometry after two IR immunizations with RV-VLP and LT-R192G, compared to a single immunization, despite a trend towards a higher response on day 4 for the B220^int^ subset, consistent with a conventional secondary antibody response. Of note, the primary and secondary responses were not different in mice immunized in the absence of adjuvant. In these conditions, a great variability was observed between mice after one or two immunizations, without any correlation between the magnitude of the B cell response analyzed by flow cytometry and the antibody response. Analysis of antibody secreting cells by Elispot in the *lamina propria* and the bone marrow may have probably shown a better correlation with the antibody response. Nevertheless, these results which, confirm previous results obtained via the intranasal route, suggest that most B cells analyzed by FCM probably do not differentiate into antibody secreting cells and could have another role in the early response. Firstly, they may be important in antigen presentation and thus impact on T cell responses. Secondly, a large proportion of the B cells analyzed were B1-a cells expressing CD5, in the primary [10] as well as in the secondary response (data not shown). B1-a cells produce natural polyspecific antibodies which most likely play a role in early protection during experimental challenge. However, natural antibodies are also potentially auto-reactive [22], especially as high frequencies are induced. Thus, we hypothesized that these cells should be regulated to avoid deleterious autoreactivity. Immunomodulation is an intrinsic property of the immune system and is partially mediated by T cells. Tregs include different subsets comprising natural and inducible CD4^+^ Tregs expressing CD25 and Foxp3 that we wanted to investigate first, notably because we hypothesized that nTregs should be involved early to control auto-immune reactivity. Quantitative analysis reflecting activation and/or proliferative responsiveness of CD4^+^CD25^+^Foxp3^+^ T cells, presumably Tregs (nTregs or iTregs), was performed using cell frequency, CD25 and Foxp3 mean fluorescence intensity in *in vitro* culture experiments in the presence of the antigen, the adjuvant or both, as this method is classically used to study T cells [19]. This allowed us to also analyze CD4^+^CD25^+^Foxp3^−^ T cells, presumably helper T cells, as well as IL-2 production. 

In these experimental conditions, for non-immunized mice, we observed a decrease in CD4^+^CD25^+^Foxp3^+^ cell frequency, from day 2 to day 4, in wells with RPMI only. This is in accordance with the fact that CD4^+^CD25^+^Foxp3^+^ Tregs are dependent on IL-2 for survival [14] and no IL-2 was produced in these wells. However, the decrease was more important in the presence of LT-R192G and both 2/6-VLP and LT-R192G. Moreover, there were major differences between the antigen and the adjuvant, as 2/6-VLP induced a weak but homogenous decrease in Foxp3 expression leading to a weak decrease in CD4^+^CD25^+^Foxp3^+^ cell frequency together with an increase in CD4^+^CD25^+^Foxp3^−^ cell percentage. This result suggests that 2/6-VLP could be a negative regulator of Foxp3 expression by nTregs, however this hypothesis has to be confirmed. Unlike 2/6-VLP, LT-R192G induced a major decrease of CD4^+^CD25^+^Foxp3^+^ cells, that was only transiently associated with an increase in the CD4^+^CD25^+^Foxp3^−^ population. Another important difference between 2/6-VLP and LT-R192G was the decrease in CD25 expression induced by LT-R192G on both Foxp3^+^ and Foxp3^−^ cells. This effect was observed at the dose of 0.01 µg/mL of LT-R192G with a maximum at the dose of 0.5–1 µg/mL, reflecting the power of this molecule. We excluded the possibility that LT-R192G had a toxic effect on CD4^+^CD25^+^Foxp3^+^ cells for several reasons: firstly, LT-R192G is devoid of detectable *in vitro* ADP-ribosyltransferase activity [2]; secondly, the effect of LT-R192G on CD4^+^CD25^+^Foxp3^+^ cells is different between a first contact and a recall *in vitro*; thirdly, no decrease in CD4^+^CD25^+^Foxp3^−^ T cells was observed. We suggest that LT-R192G may induce selective CD4^+^CD25^+^Foxp3^+^ cell apoptosis during a first contact. Others have reported that LT induces apoptosis in mature lymphocytes *in vitro and* CD8^+^ T cells were more susceptible than were CD4^+^ T cells [1]. In the same manner, CD4^+^CD25^+^Foxp3^+^ Treg cells may be more susceptible to apoptosis induced by LT-R192G than CD4^+^CD25^+^Foxp3^−^ helper T cells. Thus, the effect of LT-R192G observed here on the CD4^+^CD25^+^Foxp3^+^ subpopulation may be the result of selective apoptosis because of a higher susceptibility, compared with CD4^+^Foxp3^−^ T cells. One hypothesis could be that CD4^+^CD25^+^Foxp3^+^ cells first decrease CD25 expression before cell death by apoptosis. As CD4^+^CD25^+^Foxp3^+^ cells are present at a low frequency among CD4 cells, specific labelling is necessary to show this effect. 

For mice immunized with 2/6-VLP and adjuvant, *in vitro* restimulation with the antigen showed, as expected, specific activation of CD4^+^CD25^+^Foxp3^−^ T cells as shown by the increase in CD25 expression on day 2 and in cell frequency on day 4, together with IL-2 production, these effects increasing with the dose. The activation of CD4^+^CD25^+^Foxp3^−^ T cells was associated with the activation of CD4^+^CD25^+^Foxp3^+^ T cells but with a different kinetics. Indeed, Foxp3 expression was first increased on day 2, followed on day 4 by CD25 expression and cell frequency, suggesting that activation of CD4^+^CD25^+^Foxp3^+^ T cells is delayed, which is consistent with a feedback loop regulation depending on IL-2 [19]. Moreover, these activated CD4^+^CD25^+^Foxp3^+^ T cells are probably 2/6-VLP specific iTregs. 

Of note, T cell activation was observed for all the lymphoid organs and tissues analyzed indicating that T cell migration differs from B cell migration after IR immunization, as previously shown for the IN route. Indeed B cells were not found in PP in both cases or in the spleen with the IR route, whereas T cells were found in these tissues [11,23]. This result was made possible because our model allowed us to trace both specific B and T cells. The migration of primary and memory RV specific CD8+ T cells in several non lymphoid and lymphoid tissues has also been reported previously [24]. To our knowledge, there are no published reports of such patterns of migration showing differences between B and T cells after mucosal immunization.

Unlike mice immunized with 2/6-VLP in the presence of adjuvant, no cell activation in the CD4^+^CD25^+^Foxp3^−^ population and no IL-2 production was observed for mice immunized with 2/6-VLP alone. This result is in accordance with previous results [23] observed with the IN route which also showed a lack of IL-2 production by lymphoid cells from mice immunized with 2/6-VLP alone. These results suggest that the adjuvant is necessary to induce and detect a sufficient level of memory T cells after *in vitro* restimulation. No cell activation in the CD4^+^CD25^+^Foxp3^+^ population was neither observed for mice immunized with 2/6-VLP alone, which is consistent with the fact that IL-2 is critical in CD4^+^CD25^+^Foxp3^+^ cell activation [14] and with the model of feedback loop regulation.

*In vitro* restimulation with LT-R192G showed important differences between mice immunized with 2/6-VLP and adjuvant and control mice. Indeed, whereas a decrease in CD25 MFI and in CD4^+^CD25^+^Foxp3^+^ cell frequency was observed on day 2 in both cases, it was associated with an increase in Foxp3 expression in mice immunized with 2/6-VLP and adjuvant, suggesting a specific recall of CD4^+^CD25^+^Foxp3^+^ cells induced *in vivo* (iTregs). Moreover, on day 4, CD25 MFI as well as cell frequency did not decrease anymore. Similar results were observed for mice immunized with LT-R192G alone (data not shown). Thus the major decrease in this subpopulation, observed after a first contact, may be attributed to specific apoptosis of nTregs induced by LT-R192G, whereas *in vivo* induced Tregs, specific either for LT-R192G or for 2/6-VLP, may be less susceptible to apoptosis. Moreover, Tregs specific for LT-R192G can be recalled *in vitro* as shown by the increase in Foxp3 expression and probable proliferation. 

All together, these results suggest that LT-R192G induces specific Tregs during the prime that can be recalled *in vitro* and also promotes the induction of antigen specific Tregs. 

As observed during a first contact with LT-R192G, we found a decrease in CD25 expression during a second contact. CD25 is the α chain of the IL-2 receptor and is necessary to form, with the β and γ chains, the trimeric receptor, necessary for high affinity binding of IL-2. A decrease in CD25 expression may result in impairment in Treg maintenance and/or activation and/or function. However, it has been reported recently [25] that Tregs expressing a mutant IL-2Rβ chain supported suboptimal and transient STAT5 activation by IL-2, resulting in upregulation of Foxp3 in nTregs. This observation indicates that Tregs can adapt to low IL-2 through fully inducing and maintaining Foxp3 by a minimal requirement for IL-2 dependent STAT5 activation. Here, we observed an increase in Foxp3 expression in Treg cells together with a decrease in CD25 on day 2 during the recall with LT-R192G *in vitro*. This result suggests that CD4^+^CD25^+^Foxp3^+^ cells may play a functional suppressive role despite impairment of IL-2 binding, which is consistent with the fact that Foxp3 expression depends in part on STAT5 activation. 

Finally, culture experiments in the presence of both 2/6-VLP and LT-R192G showed that LT-R192G clearly modulates specific CD4^+^CD25^+^Foxp3^−^ helper T cells as shown by the significant decrease in IL-2 production, CD25 MFI and cell frequency in wells restimulated with both 2/6-VLP and LT-R192G, compared to wells with 2/6-VLP alone. This was associated with a modulation of CD25 but not Foxp3 expression on CD4^+^CD25^+^Foxp3^+^ T cells as well as a decrease in cell frequency. One may suppose that LT-R192G specific iTregs modulates the activation of CD4^+^CD25^+^Foxp3^−^ T cells, the resulting weak IL-2 production reducing the activation of 2/6-VLP specific iTregs. Of note, the effect of LT-R192G on CD25 expression is consistent with previous results which showed a decrease in CD25 expression on activated CD4^+^ T cells by anti-CD3 or phytohemagglutinin [26,27], though in the previous experiments, CT and a mitogen or anti-CD3 were used. Moreover, we found that LT-R192G also modulates CD4^+^ T cells, both Foxp3^+^ and Foxp3^−^, activated by conA (data not shown). 

Thus, LT-R192G which, like LT or CT, is a potent mucosal adjuvant for inducing immune B and T cell responses, is also able to induce specific Tregs and promote iTregs against 2/6-VLP. In accordance with our results, Lavelle *et al.* have shown that CT induces Tr1 T cells that suppressed IFNγ production after SC immunization [28]. We propose that Tregs induced in our model could modulate not only B cell expansion during the secondary response, but also memory T cell activation to avoid auto-immunity as well as inflammation. Of note, one cannot exclude that CD4^+^CD25^+^Foxp3^−^ cells contain regulatory T cells, such as Tr1 which express CD25 after activation [29]. 

Induction of CD4^+^CD25^+^Foxp3^+^ Tregs has been reported for the B subunit of CT when administered in a conjugated form to OVA, thus resulting in mucosal tolerance [30]. The importance of B cells in the induction of Tregs in this context has been reported. Moreover, it has been reported recently [31] that a subpopulation of B cells expressing CD1d(hi) and CD5 is a unique subset of potent regulatory B cells involved in auto-immunity and inflammatory models, IL-10 being a key component in B-cell-mediated immune regulation [32]. So, the high B cell expansion observed in our model during the primary response, which may have an important role in antigen presentation and in producing natural polyreactive antibodies, critical for protection against infection, could also play a role in limiting the inflammatory mechanisms and autoimmunity that could be induced in the context of a strong response to the presence of adjuvant. B cells may exert a regulatory role either by themselves and/or by inducing Tregs. 

Finally, in this work, we showed that CD4^+^CD25^+^Foxp3^+^ T cell activation by RV 2/6-VLP was observed only in the context of a high immune response, when adjuvant was used for immunization. This is consistent with a feedback loop regulation, *i.e.*, a specific activation of CD4^+^CD25^+^Foxp3^−^ T helper cells which secrete IL-2 that makes CD4^+^CD25^+^Foxp3^+^ Tregs proliferate and in turn control helper T cells [19]. The impact of LT-R192G observed *in vitro* on cell frequency, CD25 and Foxp3 expression suggests that this adjuvant plays an important role in modulating the CD4^+^CD25^+^Foxp3^+^ Treg subpopulation. Although we could not demonstrate a direct effect of LT-R192G *in vivo*, we argue, on the basis of *in vitro* experiments, that the first contact *in vivo* with LT-R192G in a context of low IL-2, may induce countersuppression by decreasing Treg numbers and/or activation, thus allowing naïve B and T cell activation as well as B1-a cell expansion, which would account for the adjuvant effect. Tregs should be also induced by both the antigen and the adjuvant and may control immune responses during the secondary response. Specific B cells may also play a role either as regulatory B cells or by inducing Tregs. Consistent with our hypothesis, we found that immunization during the boost with 2/6-VLP alone (*i.e.*, without adjuvant), did not induce lower Ab responses compared to a boost with LT-R192G (data not shown). These findings ask for the need to use the adjuvant during the boost, the amplitude of the memory antibody response being most likely determined during the prime. 

## 5. Conclusions

The results confirmed those obtained by the IN route for B cell responses. In addition, they showed an important effect of the adjuvant on CD4^+^CD25^+^Foxp3^+^ Tregs *in vitro*, which was different in the first and second contact, as well as a negative regulation of 2/6-VLP specific CD4^+^CD25^+^Foxp3^+^ and Foxp3^−^ T cells. These results will have to be completed by testing other antigens and adjuvants and should be considered for the design of mucosal vaccination protocols.
